# Utilizing a Diffractive Focus Beam Shaper to Enhance Pattern Uniformity and Process Throughput during Direct Laser Interference Patterning

**DOI:** 10.3390/ma15020591

**Published:** 2022-01-13

**Authors:** Mikhael El-Khoury, Bogdan Voisiat, Tim Kunze, Andrés Fabián Lasagni

**Affiliations:** 1 Institute for Manufacturing Technology, Technische Universität Dresden, George-Baehr-Str. 3c, 01069 Dresden, Germany; bogdan.voisiat@tu-dresden.de (B.V.); andres_fabian.lasagni@tu-dresden.de (A.F.L.); 2 Fusion Bionic GmbH, Winterbergstraße 28, 01277 Dresden, Germany; tim.kunze@fusionbionic.com; 3 Fraunhofer-Institut für Werkstoff- und Strahltechnik IWS, Winterbergstr. 28, 01277 Dresden, Germany

**Keywords:** direct laser interference patterning, fundamental beam mode shaper, top-hat beam profile, filling-factor, throughput, homogeneity

## Abstract

Uniform periodic microstructure formation over large areas is generally challenging in Direct Laser Interference Patterning (DLIP) due to the Gaussian laser beam intensity distribution inherent to most commercial laser sources. In this work, a diffractive fundamental beam-mode shaper (FBS) element is implemented in a four-beam DLIP optical setup to generate a square-shaped top-hat intensity distribution in the interference volume. The interference patterns produced by a standard configuration and the developed setup are measured and compared. In particular, the impact of both laser intensity distributions on process throughput as well as fill-factor is investigated by measuring the resulting microstructure height with height error over the structured surface. It is demonstrated that by utilizing top-hat-shaped interference patterns, it is possible to produce on average 44.8% deeper structures with up to 60% higher homogeneity at the same throughput. Moreover, the presented approach allows the production of microstructures with comparable height and homogeneity compared to the Gaussian intensity distribution with increased throughput of 53%.

## 1. Introduction

In recent years, laser surface texturing (LST) has proven to be a suitable tool for producing various surfaces with controllable topography, leading to improved surface properties, such as wettability [1,2] and self-cleaning [3], tribology [4] and antifouling properties [5]. Nowadays, out of the available LST methods, Direct Laser Interference Patterning (DLIP) has arisen as an innovative and effective tool for high throughput surface micro-structuring [6,7,8,9]. This technique enables a direct fabrication of flexible and perfect periodic surface patterns with a well-defined long-range order based on the interference principle [10,11,12,13,14,15]. In addition, it has been shown that the number of interfering laser beams, their geometrical arrangement, individual angles, phase and polarization influence the shape of the interference pattern as well as its typical repetitive distance (spatial period) [16,17]. Due to the flexibility to achieve highly complex patterns in a one-step process, DLIP is especially interesting for industrial applications. Moreover, no chemicals, post-treatments or vacuum conditions are required, making it an eco-friendly, fast and cost-optimized process [12].

**Figure 1:**
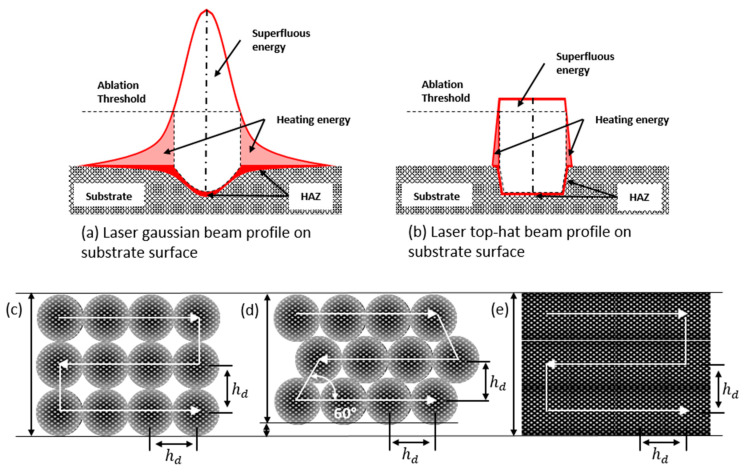
Laser energy contribution to material ablation and formation of Heat affected Zone (HAZ) when (**a**) Gaussian and (**b**) top-hat beams are utilized Illustration of structured area coverage during DLIP with (**c**) square and (**d**) hexagonally distributed Gaussian laser spots as well as (**e**) top-hat shaped laser beam. h_d_ denotes a hatch distance between two consecutive pulses.

During DLIP processing, the material interacts with the laser radiation predominantly at the positions corresponding to the interference maxima, inducing various metallurgical processes such as melting, ablation and recrystallization [18,19]. During nanosecond-pulsed laser processing of metals, the structuring mechanism is mainly based on recoil vapour pressure and Marangoni convection, that have an effect on the overall picture of melt flow [20,21]. Since the local intensity at the interference maxima positions is directly related to the intensity distribution in the laser beam profile, the use of a round Gaussian beam leads to inhomogeneous surface textures due to the non-uniform intensity distribution of the input laser beam [22,23]. However, most commercial lasers provide beams with Gaussian (TEM00) intensity distribution. This intensity profile preserves its distribution during propagation, and it can be focused to a diffraction-limited spot. Furthermore, the energy distribution of the Gaussian beam gradually decreases from the center to the boundary of the laser spot. Consequently, the spot area limited by a beam diameter (at 1/e^2^ level) includes only 86.5% of the laser beam energy, and the intensity at the boundary is only 13.5% of the peak intensity [23]. Moreover, previous investigations showed that only 36.8% of the pulse energy is used efficiently at the focal position for a Gaussian beam [24,25]. In particular, the energy per unit of area (fluence) at the tails can be much lower than the ablation threshold leading to undesired heating effects of the surface surrounding the laser spot without any ablation (see Figure 1a). In addition, the excess energy located at the center of the beam can lead to uncontrolled melting (over melting), which in turn affects the microstructure quality. Consequently, nearly two-thirds of the used laser power of a Gaussian beam is potentially wasted during laser micro-structuring process.

Another disadvantage of the Gaussian beam profile is the footprint of the ablated zone, which has a round shape and causes both an inhomogeneous profile along the processing line and a low fill-factor [26] (see Figure 1c,d). This is usually circumvented by an increase of the pulse-to-pulse overlap that leads to an improved pattern homogeneity but at the same time to lower throughputs and an increased Heat Affected Zone (HAZ) [23,26,27]. Different process strategies have been already developed to improve the uniformity of the produced structures and process throughput. For instance, in the work of B. Voisiat [7], the ablation rate in interference patterning was improved by developing a laser source providing directly a top-hat profile. In this way, the process strategy shown in Figure 1e can be implemented. However, for creating the interference pattern, an image optic system had to be developed which means that for producing different pattern geometries and sizes, certain optical elements have to be replaced. In consequence, the mentioned strategy cannot be implemented with standard laser sources providing Gaussian beams. In recent research, the concept of implementing a fundamental beam-mode shaper (FBS) [28,29] applied to a four-beam DLIP setup was introduced [30]. Although the possibility of producing interference patterns within a top-hat envelope was demonstrated, up to now, this configuration was not utilized to process any kind of material and its advantages in terms of increased throughput was not experimentally demonstrated. 

In this context, this study presents the usage of FBS in combination with a DLIP optical configuration to treat flat metallic surfaces. The interference patterns produced by the symmetrical four-beam DLIP setup with Gaussian and top-hat intensity distributions are compared. The impact of the laser fluence and hatch distance on the process throughput, the filling factor of the structured area and the resulting microstructure height and the height error across the structured surface are analysed and compared.

## 2. Materials and Methods

### 2.1. Sample Preparation

The DLIP laser texturing experiments were performed on flat samples of 1.4301 stainless steel (AISI 304). This material was chosen due to its wide application fields, including sinks, kitchen equipment such as pans, tubing etc. It has to be mentioned that the objective of this work relies on demonstrating how it is possible to improve the homogeneity of structured surfaces as well as to increase throughput. Therefore, the selection of this material is related only to demonstration purposes. Samples have a thickness of 0.7 mm and average surface roughness (Ra) of 52 nm. The substrates were used as received.

### 2.2. Principle of a Fundamental Beam-Mode Shaper

The fundamental beam-mode shaper is a specially designed diffractive optical element introduced in front of the focusing lens, which converts the input Gaussian beam distribution into a square-shaped top-hat intensity distribution at the focal plane. The FBS beam shaper typifies a kind of phase plate with a continuous and smooth phase relief profile which redistributes irradiance and phase of the wave front [23,29]. The depth of focus for the reshaped beam FBS element is close to the Rayleigh length of the Gaussian beam focused with the same lens [23,29]. The FBS element has a high conversion efficiency of 95% in an area limited by 1/e^2^ level, where only 5% of the laser energy is dissipated in the tail region. The tolerance of areal uniformity of the top-hat profile is ±2.5%. The FBS approach requires specific input laser beam parameters, such as high beam quality (TEM00, M^2^ < 1.4) and a particular beam diameter of 4 mm with ±5% tolerance [23,28].

### 2.3. Nanosecond Four-Beam-DLIP Setup and Process Strategy

**Figure 2:**
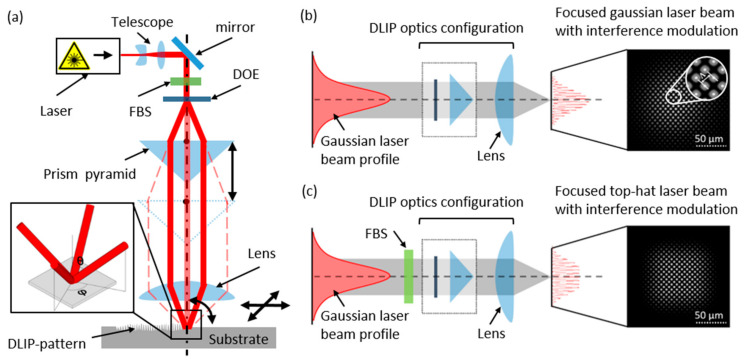
(**a**) Schematic representation of the experimental setup for structuring dot-like pattern using a DOE-based DLIP optical configuration combined with an FBS element; the inset at the sample surface shows the sketch of four symmetrically distributed interfering sub-beams. The incidence angle *θ* is the same for all four beams. The azimuthal angle *ϕ* between each consecutive beam is 90° and the spatial period can be calculated by $\Lambda_{4}=\lambda /(\sqrt{2}\cdot \sin\theta )$, where λ is a laser wavelength. Representation and measurement of the interference pattern distribution (with a beam profiling camera) for the setup without (**b**) and with (**c**) the FBS element.

The laser experiments were carried out using a four-beam interference setup, producing interference patterns with a dot-like morphology. A schematic representation of the experimental DLIP setup (Fraunhofer IWS, Dresden, Germany) [31] is shown in Figure 2a, which includes a nanosecond (ns)-pulsed laser system (Laser Tech 1053 Advanced, Laser Export) operating at a wavelength of 1053 nm with a maximum average output power of 4 W (at 4 kHz repetition rate) and 6 ns pulse duration. The experimental setup also includes a compact DLIP optical configuration, where the main beam is split into four identical sub-beams using a diffractive optical element (DOE). Then, the beams are parallelized by a pyramid (with an apex angle of 138.4°) and finally focused and superposed by using a condenser lens with a focal distance of 100 mm. The four sub-beams are symmetrically distributed around the optical axis, as depicted in Figure 2a. The FBS beam shaper (TOPAG Lasertechnik GmbH, Darmstadt, Germany) was integrated prior to the four-beam DLIP optical configuration [30] and a confocal telescope with a 4× magnification was used to provide an input beam at the FBS element with a diameter of 4.0 mm. The resulting interference intensity pattern consists of periodically distributed peaks (DLIP pattern) with square symmetry, as shown in Figure 2b,c. The laser fluence (or energy density), defined as the laser energy deposited per unit of area, is one of the most important parameters that control the morphology and quality of the periodic patterns as has been shown in other studies [21,27]. In this work, the laser fluence Fp was varied by adjusting the laser power between 70 and 100% (corresponding to 0.95 and 1.93 W, respectively). In this way, the range of used laser fluences can be calculated being from 2.10 J/cm^2^ up to 2.97 J/cm^2^. The beam radius, determined using the D-squared method, was 64.7 μm [32]. To structure larger areas, the sample was moved using XY-stages (PRO Series, Aerotech Ltd., Ramsdell, UK) The arrangement of the pulse positioning during the structuring process is illustrated schematically in Figure 1c–e. In this way, the laser pulses were shifted and overlapped in the x and y-directions in such a way that the hatch distance (h_d_) was always kept as a multiple of the spatial period (Λ_4_ = 6.5 µm) to guarantee a well-defined periodic structure. Consequently, h_d_ values of 71.5 µm and 104.0 µm result when considering 6 and 11 times the spatial period, respectively. Furthermore, the hatch distance is a very relevant parameter for maximizing throughput as already published in [21,27]. The maximum throughput (Thr) is proportional to the hatch distance and it can be calculated by Equation (1):

Thr [cm^2^/min] = h_d_·2·f, (1)

where f is the repetition rate. In our case, the repetition rate was set to 4 kHz.

### 2.4. Surface Characterization

To describe the uniformity of the fabricated structures, two topographical parameters were used, namely the structure height error and the filling factor (short: fill-factor). The last describes the ratio between surface completely covered with microstructures and actual structured surface. For this purpose, the surface topography of the structured samples was measured using a confocal microscope (Sensofar, S Neox non-contact 3D Surface Profiler, Barcelona, Spain) utilizing a 50× magnification objective. This objective allows recording 351 µm × 264 µm areas at each measured position with lateral and vertical resolutions of 340 nm and 4 nm, respectively. Afterward, using the software MountainsMap 7.4 (Digital Surf, Besançon, France), the surface profiles of the measured topographies are extracted applying morphological filters (ISO16610-14), and the topographical 3D roughness parameters are calculated (ISO 25178-2) [27]. A morphological filter is applied on the area of interest and it is based on dilation and erosion operation, with the structuring element (SE) set to the size of the structure spatial period (Λ_4_ = 6.5 µm). By applying the dilation operation, the upper structure envelope is calculated that represents the height distribution of structure hills. In the same manner, erosion operation extracts the lower structure envelope, which represents the distribution of depths of structure valleys. Accordingly, the fabricated structure average height distribution is achieved by subtracting the lower from the upper envelopes. Referring to ISO 25178-2, the average structure height (S_mean_) and root mean square (S_q_) can be noted and structure height error can be calculated (h_err_ = (S_q_⁄S_mean_)·100%) [27]. In addition, high-magnification images were acquired using a scanning electron microscope at an operating voltage of 15 kV (JEOL Ltd., JSM 6610LV, Akishima, Tokyo, Japan). The captured images were analyzed using ImageJ 1.8.0_172 software (Java-based software developed by National Institutes of Health, Bethesda, MA, USA) [33]. The fill factor was determined by the following steps: (i) binarization (8 bit), (ii) application of a threshold (>55.20%) and (iii) pixel interpretation (number of white and black pixels) employing the Otsu method [34]. 

### 2.5. Design of Experiments

To analyze the effect of beam shaping in the DLIP process, a set of experiments were designed. The first experiments aimed to study the pattern characteristics of a single DLIP spot ablated with Gaussian and top-hat beam profiles. For this purpose, the steel surfaces were first irradiated with laser spots forming compact and regular square arrays with a fixed hatch distance h_d_ of 130 µm, this being value larger than the beam diameter of the interference region (~120 µm), in order to achieve separate ablated areas to be analyzed individually, and variable laser fluence values from 2.10 up to 2.97 J/cm^2^. The effect of beam reshaping from Gaussian to a top-hat beam was analyzed by means of the structure height, structure error and ablated spot size on a defined 130 µm × 130 µm area of interest, as mentioned in the previous section. Measurements were performed at three random positions inside the structured area. 

The second set of experiments aimed to investigate the beam shaping effect on large area structuring in terms of surface quality and process throughput. For this purpose, matrices containing structured areas of 3 mm × 3 mm in size were treated for three different process conditions: (i) Gaussian beam interference with a square distribution of ablated spots (Figure 1c); (ii) Gaussian beam interference with a hexagonal distribution of ablated spots (Figure 1d); and (iii) square-shaped top-hat beam interference pattern with a square distribution of the ablated spots (Figure 1e). In each matrix, the hatch distance between the pulses was varied from 71.5 to 104.0 µm as was the laser fluence from 2.10 up to 2.97 J/cm^2^. The effect of beam reshaping from Gaussian to a top-hat beam on large area structuring was analyzed on a defined, 351 µm × 264 µm area of interest, as described in the previous section. The measurements were performed over a defined area of interest at a random position inside the structured area.

## 3. Results and Discussion

**Figure 3:**
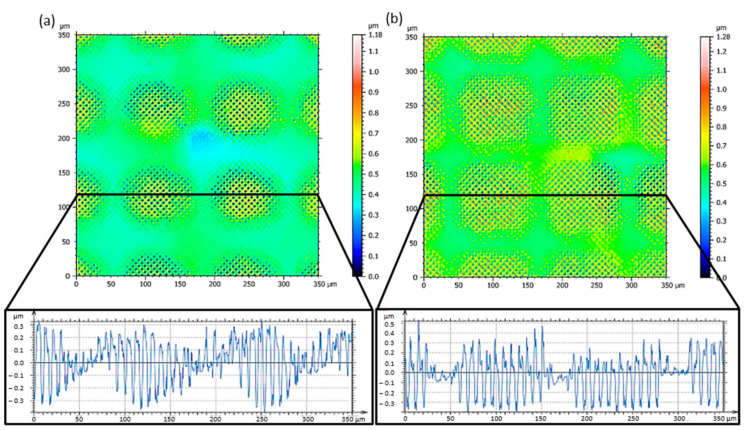
Confocal microscope images of surface topographies produced in stainless steel and corresponding surface profiles using the (**a**) Gaussian and (**b**) top-hat beam profiles. Only one laser pulse was used for each spot. The laser fluence was 2.97 J/cm^2^ and the hatch distance was 130 µm.

Confocal microscope images of surface topographies are presented in Figure 3 showing regular ordered hole-like patterns with a spatial period of Λ_4_ = 6.5 µm on the steel surface, irradiated with one pulse per spot at a laser fluence of 2.97 J/cm^2^ using both Gaussian and top-hat beam profiles. In Figure 3, also the surface profiles of the produced topographies are shown, which follow the laser beam intensity distribution shown in Figure 2b,c, respectively (beam profiling camera measurements). 

The obtained results for both configurations are clearly different. In case of the DLIP setup without the FBS element, due to the Gaussian distribution of the laser beam intensity, the ablated spots are rounded and the structure depth decreases gradually, going from the center to the edges of the spot (Figure 3a). In contrast, the patterns produced with the top-hat intensity profile exhibit approximately the same structure depth all over the square shaped spot (Figure 3b). Besides material ablation during nanosecond DLIP processing, redeposition of the molten material driven by Marangoni convection and recoil vapor pressure [20,35] takes place. It has to be also mentioned that during four-beam DLIP, all round-shaped holes are formed simultaneously at the interference maxima positions. Therefore, during the ablation process, the flow of molten material from interference maxima to minima positions results in a merging of recast material at the regions surrounding the holes, leading to structures with higher aspect ratios [36,37]. In the case of the Gaussian beam, the amount of recast material decreases near to the spot edge due to the lower laser intensities, while in the middle of the Gaussian beam (see surface profile in Figure 3a) it forms high peaks due to superfluous energy. Consequently, the inhomogeneous laser intensity leads to a reduced uniformity of the final structure. However, in the case of the top-hat beam distribution, the recast material is formed around the holes with roughly the same height (see profile in Figure 3b) due to the even intensity distribution in the beam profile.

**Figure 4:**
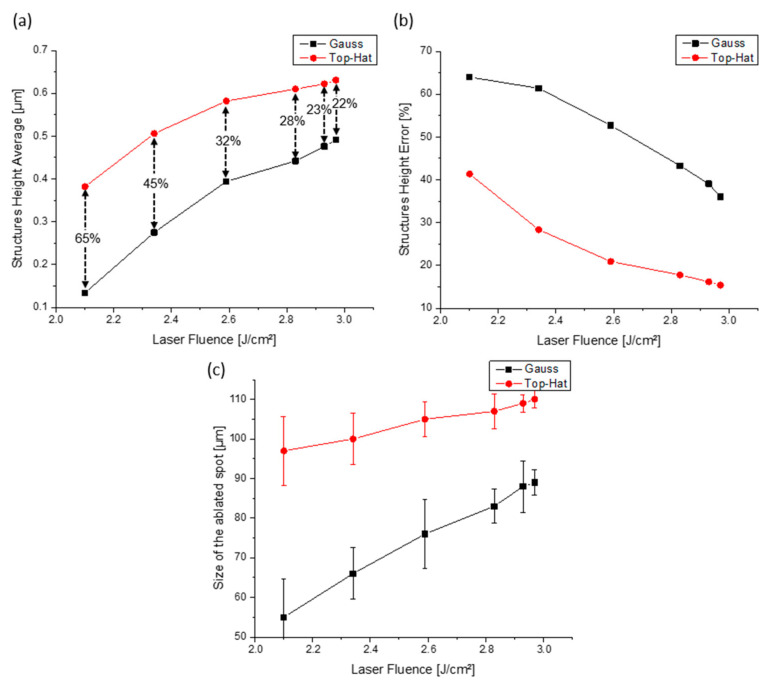
Topographical parameters of hole-like structured steel samples with both Gaussian and Top-hat beams: (**a**) average structure height, (**b**) structure height error, (**c**) size of the ablated spot at different laser fluence values. The lines serve only as a guide to the eye.

Figure 4 shows the effect of the laser fluence variation on the characteristics of topographies structured with Gaussian and top-hat laser beams. The single ablated spot structure height was calculated using the method described in the surface characterization section. The average structure height was measured over a squared-shaped area of interest of 130 µm × 130 µm centered with the ablated spot. In the case of structures fabricated using the FBS element, it was observed that the produced patterns were significantly higher for the used laser fluences compared to the standard configuration. The achieved structure mean height ranged from 0.135 ± 0.086 µm to 0.490 ± 0.177 µm and from 0.380 ± 0.155 µm to 0.630 ± 0.054 µm in the case of Gaussian and square-shaped top-hat beam profiles, respectively, when laser fluence was increased from 2.10 J/cm^2^ to 2.97 J/cm^2^ (see Figure 4a). This means that the fabricated structures using the FBS element are on average 44.8% higher, according to the comparison between the average structure’s height produced with Gaussian and top-hat beam profiles, calculated with the following equation:

(2) $$\mathrm{Structure}\;\mathrm{Height}\;\mathrm{improvement}\;\left[\%\right]=\frac{1}{n}\sum_{i=1}^{n}\frac{\left|S_{\mathrm{mean}}{}_{F_{n}}^{\mathrm{Gauss}}-S_{\mathrm{mean}}{}_{F_{n}}^{\mathrm{Top}-\mathrm{hat}}\right|}{S_{\mathrm{mean}}{}_{F_{n}}^{\mathrm{Top}-\mathrm{hat}}}\times 100\%,$$

where $S_{\mathrm{mean}}{}_{F_{n}}^{\mathrm{Gauss}}$ and $S_{\mathrm{mean}}{}_{F_{n}}^{\mathrm{Top}-\mathrm{hat}}$ are structure mean height at used laser fluence ($F_{n}\in \left[2.10\;J/{\mathrm{cm}}^{2}:2.97\;J/{\mathrm{cm}}^{2}\right]$), patterned with Gaussian and top-hat beam profiles, respectively.

Moreover, the reshaping of the beam allowed producing more uniform patterns. The structure uniformity can be described in terms of average structure height error, which is plotted in Figure 4b with respect to the applied laser fluence. It can be seen that, in the case of the square-shaped top-hat profile, the height error is always significantly smaller than in the case of the Gaussian beam profile, denoting a more uniform fabricated pattern. On average, the structure height error was 48.7% smaller for the top-hat beam and is decreasing from 41% down to 15% when laser fluence was increased from 2.1 J/cm^2^ to 2.97 J/cm^2^. Moreover, at high laser fluence, the structured surface homogeneity is improved by ~60% when the square-shaped top-hat profile was used for patterning. 

The other significant difference between the two optical configurations can be noticed in the spot size dependence on the applied laser fluence plotted in Figure 4c. For both optical configurations, the spot size depends almost linearly on the laser fluence. However, in the case of the Gaussian beam configuration, this increase is more pronounced (see slope of the fit), showing a more sensitive dependence of the spot size on the laser fluence, which is governed by the exponentially decreasing peak fluence from the center of the spot [32]. The reshaped beam’s intensity profile is constant across the spot area, with a sharp peak fluence drop around the edges. This drop also contributes to the variation of the spot size with the laser fluence. However, due to the sharpness of the intensity drop, this variation is much smaller.

**Figure 5:**
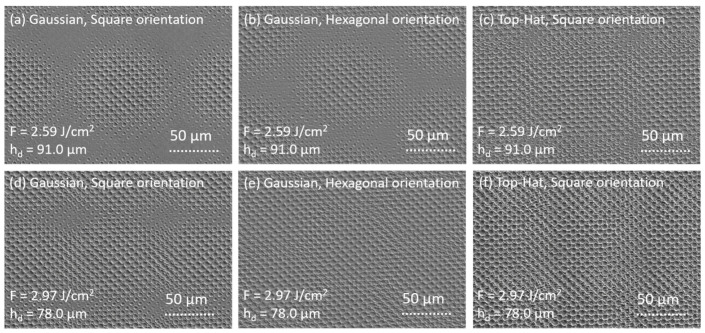
Scanning electron microscope (SEM) images of textured surfaces using the nanosecond four-beam DLIP configuration, produced on 1.4301 steel. The spatial period Λ_4_ was 6.50 μm and the following process parameters were used: (**a**–**c**) F = 2.59 J/cm^2^, h_d_ = 91.0 µm; (**d**–**f**) F = 2.97 J/cm^2^, h_d_ = 78.0 µm. The patterns were produced using (**a**,**c**,**d**,**f**) the square and (**b**,**e**) hexagonal distribution of the laser spots with (**a**,**b**,**d**,**e**) Gaussian and (**c**,**f**) top-hat configurations.

Representative SEM micrographs of the fabricated patterns for each fabrication strategy are shown in Figure 5. The patterns shown in the first row in Figure 5 are formed with a lower laser fluence of 2.59 J/cm^2^ and a relatively large hatch distance of 91.0 µm. However, the patterns in the second row in Figure 5 are formed with a maximal laser fluence of 2.97 J/cm^2^ and smaller hatch distance of 78.0 µm. It can be seen that the patterns in the first row contain non-structured areas left after the DLIP process, especially when the Gaussian beam is used (Figure 5a,b). The smallest amount of such untreated areas is visible for the case corresponding to the shaped laser beam (Figure 5c), demonstrating the benefit of a square-shaped spot for achieving better fill factors. By increasing the laser fluence and decreasing the hatch distance (Figure 5d–f), the non-structured areas also vanish in the area with hexagonal spot distribution patterned with the Gaussian beam (Figure 5e). However, in the case of square spot distribution (Figure 5d), the non-structured areas are still visible, showing the ineffectiveness of this approach in filling the area with the pattern (Figure 5a,d). In the case of square spot distribution, patterned with the shaped laser beam with increased laser fluence and decreased hatch distance (Figure 5f), the surface is completely covered with patterns and shows deeper structures.

**Figure 6:**
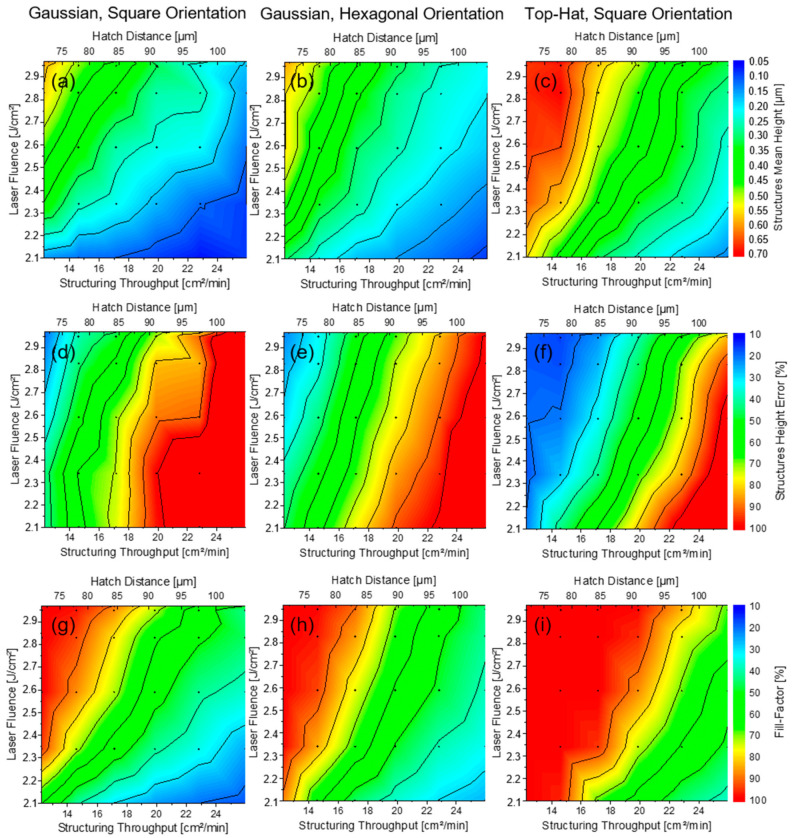
Contour plots of topography characteristics structures mean height (**a**–**c**), structures height error and (**d**–**f**), fill-factor (**g**–**i**) of the patterned surface with Gaussian ((**a**,**d**,**g**): square orientation; (**b**,**e**,**h**) hexagonal orientation) and top-hat (**c**,**f,i**) beam profiles as a function of laser fluence and hatch distance.

Each patterned area in all three matrices was analysed with a confocal microscope over a 351 µm × 264 µm area of interest and three topographical parameters were measured, namely, mean structured height, its error and the fill-factor. Figure 6 depicts the summary of the measured parameters in the form of 2D contour plots, where the parameter dependends on the applied laser fluence and hatch distance (note that the hatch distance is relevant for maximizing the fabrication throughput, as indicated by Equation (1)). 

The basic trends of the measured parameters are similar for all three patterning strategies. The mean structure height and fill factor increases with increasing laser fluence and decreasing hatch distance. However, the height error behaves with the opposite behaviour, showing the smallest values at the highest laser fluence and smallest hatch distance. The height error decreases with increasing the laser fluence and decreasing the hatch distance. The reason for the lower height errors lies in the reduction of the non-ablated area due to the higher fluences producing an enlargement of the ablated spot size. Secondly, the higher overlapping Gaussian beams provide almost constant accumulated fluence over the irradiated area, resulting in more homogeneous structures as has been previously discussed in previous work of A.I. Aguilar-Morales [21] and M. El-Khoury [27].

The main difference between the three fabrication strategies is in the slopes and absolute values of the parameter trend. The largest difference can be seen between the Gaussian and top-hat strategies, in particular for the fill-factor. The top-hat pattern’s fill-factor grows and reaches 100% of coverage area much faster than in both other cases using the Gaussian configuration (see Figure 6i). This is mainly due to the change of the footprint of the focus spot from Gaussian round geometry to square geometry of the top-hat beam profile. Round spot geometry leaves unstructured areas along the patterning path (so-called saw-tooth pattern), like in Figure 5d, as long as the pulse-to-pulse overlap is lower than 80% [38]. In addition, the redistribution of the energy from Gauss to a Top-Hat profile affects the spot’s ablation area, as was demonstrated in the first part of the experiments. This tendency demonstrates again the advantage of using square-shaped spots compared to round-shaped Gaussian beams.

The higher top-hat beam‘s filling factor also contributes to the overall higher average depth of the structures formed with the shaped laser beam (see Figure 6c), which means that the fabrication of a pattern with a specific depth can be finally produced faster with a top-hat beam. In addition, the height error of the structures fabricated with the top-hat beam setup also differs strongly from the other two structuring strategies (Figure 6d,e). In this case, the height error decreases faster with increasing laser fluence and decreasing hatch distance (see Figure 6f). Therefore, a larger process window allowing high homogeneity is possible. 

Similar advantages over the square-oriented Gaussian spots can be noted for the structures fabricated with hexagonally oriented Gaussian spots. However, the difference is much smaller in this case of structure height (Figure 6a,b) as well as structure error factor (Figure 6d,e), mainly noticeable in the fill factors (Figure 6g,h). Thus, the usage of hexagonal distribution of the Gaussian spots for patterning the large areas is another way of getting more homogeneous structures if the beam shaping option is not possible.

**Figure 7:**
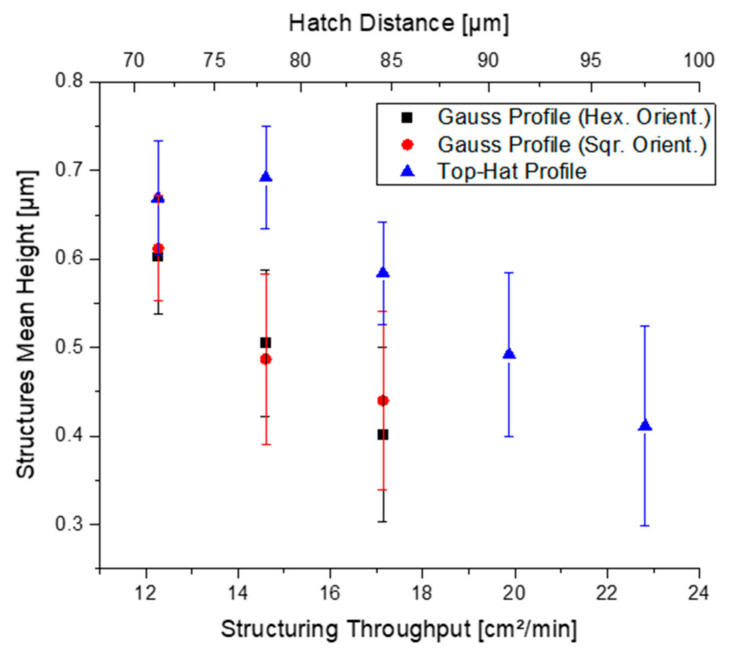
Mean height (S_mean_) and standard deviation (S_q_) of produced periodic hole-like structures having a filling factor over 80% (produced at F = 2.96 J/cm^2^, with 1.93 W of laser power and a repetition rate of 4 kHz).

Finally, to emphasize the advantage of using a reshaped top-hat laser beam in DLIP, the process throughput for fabricating periodic patterns with certain quality was calculated. As an example, periodic structures with a fill factor over 80% were produced at a laser fluence of 2.97 J/cm^2^ and the process throughput was calculated. The results are presented in Figure 7.

As can be observed, depending on the needed structure height, different throughputs are possible. For example, for a structure height of 0.5 µm, a speed of ~20.0 cm^2^/min can be reached with the top-hat beam profile, while for the Gaussian beam with either square or hexagonal arrangements, the throughput is ~14.5 cm^2^/min. Hence, it is possible to pattern the surface ~43% faster if the top-hat beam profile is used. Moreover, if higher structures are needed, the top-hat configuration allows avoiding an excess of deposited intensity on the processed surface, which is important for decreasing uncontrolled melt of the material and thus lower qualities.

## 4. Conclusions

In this study, a diffractive beam-shaping element, which applies the FBS principle to generate a square-shaped top-hat beam profile, was used in a four-beam DLIP setup. The capabilities of the FBS-DLIP system for laser surface patterning was compared with a standard configuration using a Gaussian beam. Several advantages resulted in this investigation. For example, the structure height could be increased by ~44.8% when using the top-hat configuration for the same laser fluence. Furthermore, the uniformity could be improved by ~60% due to the effective redistribution of laser intensity inside the beam. Furthermore, higher fill-factor was obtained for lower hatch distances leading to increased throughput. Finally, it was demonstrated that the productivity could be improved by ~53% (from 14.5 to 20.0 cm^2^/min), taking into consideration a periodic pattern with 0.5 µm height and over 80% filling factor.

## Data Availability

The data of this study are available from the corresponding author upon reasonable request.

## References

[1] Milles S., Soldera M., Voisiat B., Lasagni A.F. (2019). Fabrication of superhydrophobic and ice-repellent surfaces on pure aluminium using single and multiscaled periodic textures. Sci. Rep..

[2] Hauschwitz P., Jochcová D., Jagdheesh R., Cimrman M., Brajer J., Rostohar D., Mocek T., Kopeček J., Lucianetti A., Smrž M. (2020). Large-Beam Picosecond Interference Patterning of Metallic Substrates. Materials.

[3] Milles S., Soldera M., Kuntze T., Lasagni A.F. (2020). Characterization of self-cleaning properties on superhydrophobic aluminum surfaces fabricated by direct laser writing and direct laser interference patterning. Appl. Surf. Sci..

[4] Grützmacher P.G., Profito F.J., Rosenkranz A. (2019). Multi-Scale Surface Texturing in Tribology—Current Knowledge and Future Perspectives. Lubricants.

[5] Helbig R., Günther D., Friedrichs J., Rößler F., Lasagni A., Werner C. (2016). The impact of structure dimensions on initial bacterial adhesion. Biomater. Sci..

[6] Lang V., Voisiat B., Lasagni A.F. (2019). High Throughput Direct Laser Interference Patterning of Aluminum for Fabrication of Super Hydrophobic Surfaces. Materials.

[7] Voisiat B., Ströbel J., Du K., Lasagni A.F. (2020). How to improve throughput in direct laser interference patterning: Top-hat beam profile and burst mode. Laser-Based Micro- and Nanoprocessing XIV 11268.

[8] Kunze T., Zwahr C., Krupop B., Alamri S., Rößler F., Lasagni A.F. (2017). Development of a scanner-based direct laser interference patterning optical head: New surface structuring opportunities. Laser-Based Micro- and Nanoprocessing XI 10092.

[9] Brodsky A., Kaplan N. (2020). Laser surface texturing using a single diffractive optical element as an alternative for direct laser interference patterning. J. Laser Appl..

[10] Nakata Y., Yoshida M., Osawa K., Miyanaga N. (2017). Fabricating a regular hexagonal lattice structure by interference pattern of six femtosecond laser beams. Appl. Surf. Sci..

[11] Indrisiunas S., Voisiat B., Žukauskas A., Račiukaitis G. (2015). Direct laser beam interference patterning technique for fast high aspect ratio surface structuring. Laser Applications in Microelectronic and Optoelectronic Manufacturing (LAMOM) XX.

[12] Lasagni A.F., Gachot C., Trinh K.E., Hans M., Rosenkranz A., Roch T., Eckhardt S., Kunze T., Bieda M., Günther D. (2017). Direct Laser Interference Patterning, 20 Years of Development: From the Basics to Industrial Applications. Laser-Based Micro- and Nanoprocessing XI.

[13] Hung Y.-J., Chang H.-J., Chang P.-C., Lin J.-J., Kao T.-C. (2017). Employing refractive beam shaping in a Lloyd’s interference lithography system for uniform periodic nanostructure formation. J. Vac. Sci. Technol. B.

[14] Lechthaler B., Fox T., Slawik S., Mücklich F. (2020). Direct laser interference patterning combined with mask imaging. Opt. Laser Technol..

[15] Müller D.W., Fox T., Grützmacher P.G., Suarez S., Mücklich F. (2020). Applying Ultrashort Pulsed Direct Laser Interference Patterning for Functional Surfaces. Sci. Rep..

[16] Indrišiūnas S., Voisiat B., Gedvilas M., Raciukaitis G. (2017). New opportunities for custom-shape patterning using polarization control in confocal laser beam interference setup. J. Laser Appl..

[17] Lasagni A.F. High-speed surface structuring using Direct Laser Interference Patterning—Fundamentals, applications and technology transfer. Proceedings of the LPM2017—The 18th International Symposium on Laser Precision Microfabrication.

[18] Mücklich F., Lasagni A.F., Daniel C. (2005). Laser interference metallurgy—periodic surface patterning and formation of intermetallics. Intermetallics.

[19] Lasagni A.F. (2007). Advanced Design of Periodical Structures by Laser Interference Metallurgy in the Micro/Nano Scale on Macroscopic Areas.

[20] Volkov A.N., Zhigilei L.V. (2017). Melt dynamics and melt-through time in continuous wave laser heating of metal films: Contributions of the recoil vapor pressure and Marangoni effects. Int. J. Heat Mass Transf..

[21] Aguilar-Morales A.I., Alamri S., Kunze T., Lasagni A.F. (2018). Influence of processing parameters on surface texture homogeneity using Direct Laser Interference Patterning. Opt. Laser Technol..

[22] Bischoff C., Völklein F., Schmitt J., Rädel U., Umhofer U., Jäger E., Lasagni A.F. (2019). Design and Manufacturing Method of Fundamental Beam Mode Shaper for Adapted Laser Beam Profile in Laser Material Processing. Materials.

[23] Raciukaitis G. (2011). Laser Processing by Using Diffractive Optical Laser Beam Shaping Technique. J. Laser Micro/Nanoeng..

[24] Du K. (2009). Thin layer ablation with lasers of different beam profiles—Energy efficiency and over filling factor. Laser-Based Micro- and Nanopackaging and Assembly III.

[25] Du K. (2010). “Green processing” of thin film with top-hat lasers and applications in photovoltaic. Laser-Based Micro- and Nanopackaging and Assembly IV.

[26] Rung S., Barth J., Hellmann R. (2014). Characterization of Laser Beam Shaping Optics Based on Their Ablation Geometry of Thin Films. Micromachines.

[27] El-Khoury M., Voisiat B., Kunze T., Lasagni A.F. (2020). Prediction of Optimum Process Parameters Fabricated by Direct Laser Interference Patterning Based on Central Composite Design. Materials.

[28] Katz S., Kaplan N., Grossinger I. (2018). Using Diffractive Optical Elements-DOEs for beam shaping—Fundamentals and applications. Opt. Photonik.

[29] Bischoff C., Jäger E., Umhofer U. (2015). Beam Shaping Optics for Process Acceleration-Increasing the productivity of laser micromachining. Laser Tech. J..

[30] El-Khoury M., Voisiat B., Kunze T., Lasagni A.F. (2018). Utilizing Fundamental Beam-Mode Shaping Technique for Top-Hat Laser Intensities in Direct Laser Interference Patterning. J. Laser Micro/Nanoeng..

[31] Roch T., Benke D., Lasagni A.F. (2017). Method and Arrangement for Forming a Structuring on Surfaces of Components by Means of a Laser Beam. U.S. Patent.

[32] Liu J.M. (1982). Simple technique for measurements of pulsed Gaussian-beam spot sizes. Opt. Lett..

[33] Rasband W.S. ImageJ, U.S. National Institutes of Health, Bethesda, Maryland, USA, 1997–2018. https://imagej.nih.gov/ij/.

[34] Nicolò M., Rosa R., Musetti D., Musolino M., Saccheggiani M., Traverso C.E. (2002). Choroidal Vascular Flow Area in Central SerousChorioretinopathy Using Swept-Source Optical CoherenceTomography Angiography. Investig. Ophthalmol. Vis. Sci..

[35] El-Khoury M., Alamri S., Voisiat B., Kunze T., Lasagni A.F. (2020). Fabrication of hierarchical surface textures using multi-pulse direct laser interference patterning with nanosecond pulses. Mater. Lett..

[36] Voisiat B., Zwahr C., Lasagni A.F. (2019). Growth of regular micro-pillar arrays on steel by polarization-controlled laser interference patterning. Appl. Surf. Sci..

[37] Fu Y., Soldera M., Wang W., Voisiat B., Lasagni A.F. (2019). Picosecond Laser Interference Patterning of Periodical Micro-Architectures on Metallic Molds for Hot Embossing. Materials.

[38] Homburg O., Völkermeyer F., Toennissen F., Ganser H., Mitra T. High-precision Gaussian-to-tophat beam transformation improves structure quality and speed in micro-machining. Proceedings of the Fourth International WLT-Conference Lasers in Manufacturing, LIM 2007.

